# Optimum binary cut-off threshold of a diagnostic test: comparison of different methods using Monte Carlo technique

**DOI:** 10.1186/s12911-014-0099-1

**Published:** 2014-11-25

**Authors:** Gilbert Reibnegger, Walter Schrabmair

**Affiliations:** Institute of Physiological Chemistry, Center of Physiological Medicine, Medical University of Graz, A-8010 Graz, Austria

## Abstract

**Background:**

Using Monte Carlo simulations, we compare different methods (maximizing Youden index, maximizing mutual information, and logistic regression) for their ability to determine optimum binary cut-off thresholds for a ratio-scaled diagnostic test variable. Special attention is given to the stability and precision of the results in dependence on the distributional characteristics as well as the pre-test probabilities of the diagnostic categories in the test population.

**Methods:**

Fictitious data sets of a ratio-scaled diagnostic test with different distributional characteristics are generated for 50, 100 and 200 fictitious “individuals” with systematic variation of pre-test probabilities of two diagnostic categories. For each data set, optimum binary cut-off limits are determined employing different methods. Based on these optimum cut-off thresholds, sensitivities and specificities are calculated for the respective data sets. Mean values and SD of these variables are computed for 1000 repetitions each.

**Results:**

Optimizations of cut-off limits using Youden index and logistic regression-derived likelihood ratio functions with correct adaption for pre-test probabilities both yield reasonably stable results, being nearly independent from pre-test probabilities actually used. Maximizing mutual information yields cut-off levels decreasing with increasing pre-test probability of disease. The most precise results (in terms of the smallest SD) are usually seen for the likelihood ratio method. With this parametric method, however, cut-off values show a significant positive bias and, hence, specificities are usually slightly higher, and sensitivities are consequently slightly lower than with the two non-parametric methods.

**Conclusions:**

In terms of stability and bias, Youden index is best suited for determining optimal cut-off limits of a diagnostic variable. The results of Youden method and likelihood ratio method are surprisingly insensitive against distributional differences as well as pre-test probabilities of the two diagnostic categories. As an additional bonus of the parametric procedure, transfer of the likelihood ratio functions, obtained from logistic regression analysis, to other diagnostic scenarios with different pre-test probabilities is straightforward.

**Electronic supplementary material:**

The online version of this article (doi:10.1186/s12911-014-0099-1) contains supplementary material, which is available to authorized users.

## Background

Evaluation of diagnostic tests is an important issue in medical disciplines. Best known is the analysis of simple diagnostic test situations which can be represented by means of a 2 × 2-contingency table: one dimension of such a table is defined by two diagnostic categories (e.g., “non-diseased” versus “diseased”), and the second dimension represents the dichotomous test result (e.g., “normal” versus “pathological”). According to its importance, there is a large literature on the subject. A recent series of review articles presents an excellent overview covering all relevant theoretical and practical aspects of the subject [1-4].

An interesting way of evaluating diagnostic tests is provided by information theory [5-9], and an alternative elegant way of dealing with multiple, variably scaled diagnostic variables, has been suggested in 1982 by Albert [10]: he demonstrated that logistic regression analysis can be employed to compute likelihood ratio functions which, in analogy to the well-known likelihood ratio obtained from a simple 2 × 2-contingency table, are useful to compute post-test probability functions of the diagnostic categories investigated. A critical step when applying logistic regression results for the computation of likelihood ratio functions is a correction according to the pre-test probabilities of the diagnostic categories actually used for the regression procedure [10]. A combination of Albert’s findings with a generalization of the computation of post-test probabilities for more than two diagnostic categories [11,12] was demonstrated [13].

In an attempt to (1) direct new awareness to Albert’s time-honoured but nevertheless most relevant results regarding the use of logistic regression analysis in clinical chemistry, and to (2) compare logistic regression analysis with other methods for dividing patients into those with low versus those with high risk of being “diseased”, we here present the results of Monte-Carlo simulation studies. Specifically, for a diagnostic dilemma (“diseased” versus “non-diseased”) we simulate data sets for a fictitious diagnostic variable x with different pre-specified distributional characteristics for the two diagnostic categories. Then, we search for the optimum cut-off threshold of x including the following methods:

- maximizing the mutual information of the respective 2 × 2-contingency table obtained by systematically varying a binary cut-off threshold for the diagnostic variable x
- maximizing the Youden index (Youden index = sensitivity + specificity −1) by systematically varying a binary cut-off threshold for the diagnostic variable x
- performing a logistic regression analysis on the problem and searching the value of the diagnostic variable x for which the logistic regression-derived likelihood ratio (LR) function, properly corrected for the pre-test probabilities of the diagnostic categories used for the regression procedure, attains unity (i.e., the test value at which the post-test probabilities of the diagnostic categories equal the pre-test probabilities).

Major results of the simulations investigated are, for each of these three statistical procedures, the respective optimum cut-off values as well as their associated sensitivities and specificities. Besides mean values of these quantities of central interest, important “by-products” of the Monte-Carlo approach are their SD observed over the repetitive computer experiments.

We perform such calculations for four scenarios using different distributional characteristics underlying the computer-generated test data. Besides employing different sample sizes, as the most important additional control variable pre-test probabilities of disease [P(D)] are systematically varied over a wide range (from 0.10 to 0.90).

With these Monte-Carlo simulation experiments we attempt to answer the following research questions:

- How well are the two non-parametric methods (maximizing mutual information, maximizing Youden index) and the parametric method (LR technique based on logistic regression analysis) suited for determining optimum binary cut-off levels of a ratio-scaled diagnostic test, given different distributional characteristics of test data, and how well do the results of the three methods agree with the theoretical crossing points of the distribution functions underlying the two diagnostic categories?
- How do total numbers of test data and their composition in terms of pre-test probabilities of the two diagnostic categories influence the results?
- Which of the techniques yields the most precise estimates in terms of the resulting SD values of the Monte Carlo simulation runs?

## Methods

All computations are done using the commercially available computer software MATHEMATICA, version 9, by Wolfram Research, Inc., Champaign, IL, USA.

First, for the categories “no disease” and “disease”, according to P(D) chosen, fictitious patient data sets are generated using the random number generator of MATHEMATICA in combination with one out of many possible distribution functions: thus, for both diagnostic categories, fictitious data of a ratio-scaled diagnostic variable are generated following the chosen distribution functions. We choose total numbers of fictitious data sets of 50, 100 and 200, and we assume pre-test probabilities of category “disease” [P(D)] increasing from 0.10 to 0.90 in steps of 0.10. We simulate four different diagnostic scenarios:

- Scenario 1: The lognormal distribution with mean value 2.0 and standard deviation 0.4 is assumed for the “healthy” category, and with mean value 2.5 and standard deviation 0.3 for the “diseased” category.
- Scenario 2: The chi-square distribution with 7 degrees of freedom is assumed for the “healthy” category, and with 10 degrees of freedom for the “diseased” category.
- Scenario 3: The inverse gamma distribution with shape parameter 6.0 is assumed for the “healthy” category and 3.0 for the “diseased” category. The scale parameter is set to 20.0 for both categories.
- Scenario 4: The chi-square distribution with 6 degrees of freedom is assumed for the “healthy” category; for the “diseased” category, the Weibull distribution is chosen with shape parameter 10.0 and scale parameter 20.0.

Using the MATHEMATICA function FindRoot, we obtain the following crossing points for the distribution functions of the two diagnostic categories: Scenario 1, *x* = 9.20041; Scenario 2, *x* = 7.47228; Scenario 3; *x* = 5.10873; and Scenario 4, *x* = 13.4333. Differences from these values define the bias of the actually detected mean cut-off levels.

Analyses done on each data set include:

- “Empirical” determination of the cut-off value at which mutual information is maximum (“Mutual information method”): the cut-off value is systematically varied over the range of all test values by increments of 1.0, and that cut-off value is searched for which the resulting 2 × 2-contingency table produces the maximum mutual information.
- Determination of the cut-off value at which Youden index is maximum. In the following, we shall designate this method as “Youden index method”. The cut-off value is systematically varied over the range of all test values by increments of 1.0, and that cut-off value is searched for which the resulting 2 × 2-contingency table produces the maximum Youden index.
- Logistic regression analysis and calculation of the LR function (with proper correction for P(D) . Determination of the test values for which the LR functions become equal to unity (“LR method”). Briefly, logistic regression analysis on a data set for N fictitious “individuals” yields a linear predictor function α0 + α1 x, where x is the test result. The parameters α0 and α1 denote the intercept and the slope of the linear predictor. The linear predictor must be corrected for the pre-test probabilities of the diagnostic categories in order to yield the corrected linear predictor (equal to the natural logarithm of the LR function [10]):
  $$ \mathrm{l}\mathrm{o}{\mathrm{g}}_{\mathrm{e}}\left(\mathrm{L}\mathrm{R}\right) = \upalpha 0 + \upalpha 1\cdot \mathrm{x}\hbox{-} \mathrm{l}\mathrm{o}{\mathrm{g}}_{\mathrm{e}}\left[\frac{\mathrm{P}\left(\mathrm{D}\right)}{1\hbox{-} \mathrm{P}\left(\mathrm{D}\right)}\right], $$
  the argument of the logarithm on the right side of the equation being the pre-test odds.

Thus, for each data set, according to each of these three methods, three cut-off limits as well as their associated sensitivities and specificities are computed as main results.

These analyses are repeated 1000 times in order to get not only estimates of these quantities of interest, but also “empirical” estimates for their SD values.

Additionally, the effects of P(D) on the parameters α0 and α1 as well as on the properly corrected intercept parameter and, hence, on the post-test probabilities computed thereof under different diagnostic situations, are demonstrated for a specific example.

For convenience, we supply the MATHEMATICA documents necessary to reproduce our results: Additional file 1 (help.docx) gives a short explanation how to use the MATHEMATICA notebooks monte_carlo_SDev.nb (Additional file 2) which performs the necessary statistical calculations as well as the Monte Carlo simulation, and distributions.nb (Additional file 3) which produces graphical visualizations of the distribution functions used, and which calculates the crossing points of the two distribution functions for the “non-diseased” and the “diseased” fictitious individuals.

## Results

### The Monte Carlo experiments

For the Monte-Carlo experiments, we use the following conditions: total numbers of fictitious “individuals” are chosen as N =50, 100 and 200.

P(D) is varied, in steps of width 0.10, between P(D) =0.10 and P(D) =0.90.

**Figure 1:**
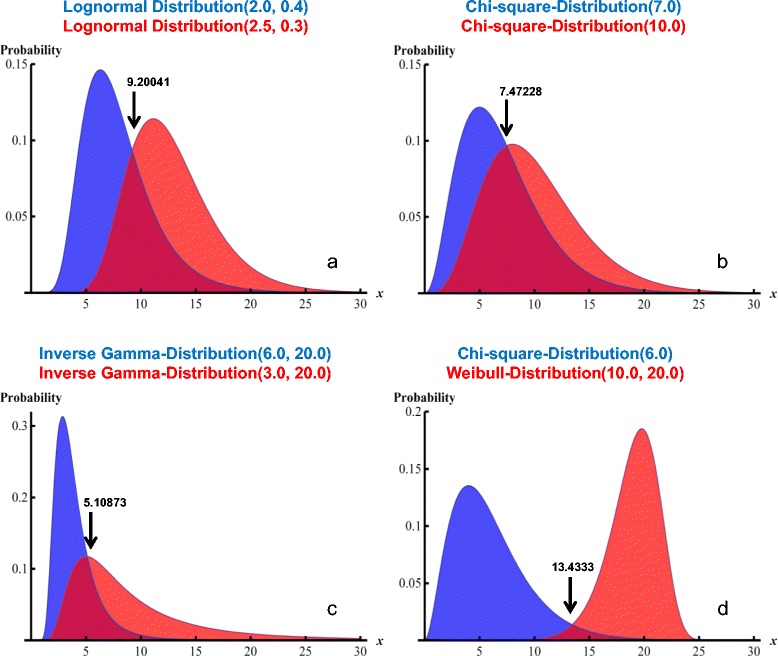
**Four different distributional scenarios.** The panels **a – d** show the visualizations of the four scenarios (see Methods section) studied. The distributional characteristics as well as the parameters determining the exact shapes of the distributions are shown in the titles of each panel. Blue: the distributions underlying the “non-diseased” category; red, the distributions underlying the “diseased” category. The arrows point at the crossing points of the distribution functions of the two diagnostic categories, and the numbers associated with arrows are the x-coordinates of these crossing points.

At each P(D), 1000 data sets, each consisting of N =50, 100 or 200 randomly chosen test values x, are generated according to the four distributional scenarios detailed in the Methods section. Figure 1 demonstrates the distribution functions underlying the four scenarios.

Each data set contains N test values, of which N × P(D) values are associated with “Disease”, and N × [1 − P(D)] values are labelled “No disease”. For each data set, the optimum cut-off threshold for x is determined by each of three different techniques (see Methods section).

Table 1 reports the ranges of the resulting mean values (and SD values) of the optimum cut-off values together with the associated sensitivities and specificities, obtained by the variation of P(D) from 0.1 to 0.9 in steps of 0.1.

**Table 1:** **Results of the Monte Carlo simulations**

	**N**		**Mutual information**		**Youden**		**Likelihood ratio**	
			**Mean**	**SD**	**Mean**	**Sd**	**Mean**	**SD**
Scenario 1 (lognormal)	50	Cut-off	8.1 - 10.4	1.7 - 2.6	9.0 - 9.7	1.2 - 1.8	9.9 - 10.1	0.5 - 1.1
		Se	0.78 - 0.86	0.17 - 0.30	0.82 - 0.91	0.10 - 0.14	0.74 - 0.76	0.08 - 0.16
		Sp	0.69 - 0.76	0.18 - 0.23	0.73 - 0.82	0.12 - 0.17	0.78 - 0.78	0.06 - 0.14
	100	Cut-off	8.1 - 10.0	1.4 - 2.2	9.2 - 9.5	1.0 - 1.4	10.0 - 10.10	0.4 - 0.7
		Se	0.84 - 0.89	0.11 - 0.19	0.81 - 0.87	0.09 - 0.13	0.74 -0.75	0.05 - 0.11
		Sp	0.65 - 0.74	0.15 - 0.22	0.72 - 0.78	0.09 - 0.15	0.78 - 0.78	0.04 - 0.10
	200	Cut-off	7.8 - 9.7	1.1 - 1.9	9.2 - 9.4	0.8 - 1.1	10.0 - 10.1	0.3 - 0.5
		Se	0.83 - 0.91	0.08 - 0.16	0.82 - 0.85	0.07 - 0.10	0.74 - 0.74	0.04 - 0.08
		Sp	0.61 - 0.72	0.12 - 0.18	0.71 - 0.75	0.08 - 0.12	0.78 - 0.78	0.03 - 0.07
Scenario 2 (chi-square)	50	Cut-off	7.4 - 9.3	3.3 - 4.1	7.5 - 8.1	1.9 - 2.7	8.2 - 8.4	0.6 - 1.1
		Se	0.67 - 0.70	0.26 - 0.35	0.69 - 0.80	0.17 - 90.21	0.59 - 0.61	0.08 - 0.17
		Sp	0.62 - 0.73	0.24 - 0.30	0.65 - 0.77	0.17 - 0.22	0.69 - 0.70	0.07 - 0.15
	100	Cut-off	7.4 - 9.0	3.1 - 4.1	7.5 - 8.0	1.5 - 2.2	8.3 - 8.4	0.4 - 0.8
		Se	0.66 - 0.71	0.23 - 0.30	0.68 - 0.76	0.14 - 0.18	0.59 - 0.61	0.06 - 0.12
		Sp	0.61 - 0.69	0.23 - 0.29	0.64 - 0.72	0.14 - 0.19	0.69 - 0.70	0.05 - 0.10
	200	Cut-off	7.0 - 8.8	2.4 - 3.9	7.5 - 7.9	1.2 - 1.8	8.3 - 8.4	0.3 - 0.5
		Se	0.67 - 0.72	0.20 - 0.27	0.67 - 0.72	0.12 - 0.16	0.59 - 0.60	0.04 - 0.09
		Sp	0.59 - 0.65	0.21 - 0.26	0.63 - 0.67	0.12 - 0.17	0.69 - 0.70	0.03 - 0.07
Scenario 3 (inverse gamma)	50	Cut-off	5.1 - 7.1	1.7 - 2.6	5.1 - 5.8	0.9 - 1.6	5.4 - 5.8	0.5 - 0.9
		Se	0.64 - 0.76	0.17 - 0.34	0.75 - 0.83	0.11 - 0.17	0.69 - 0.72	0.08 - 0.17
		Sp	0.80 - 0.87	0.15 - 0.20	0.81 - 0.90	0.11 - 0.15	0.84 - 0.85	0.05 - 0.12
	100	Cut-off	5.1 - 7.2	1.4 - 2.3	5.1 - 5.6	0.8 - 1.3	5.4 - 5.7	0.3 - 0.6
		Se	0.63 - 0.76	0.15 - 0.27	0.75 - 0.79	0.09 - 0.14	0.69 - 0.71	0.06 - 0.12
		Sp	0.81 - 0.87	0.12 - 0.19	0.81 - 0.85	0.09 - 0.13	0.84 - 0.85	0.04 - 0.08
	200	Cut-off	5.1 - 7.2	1.1 - 2.2	5.1 - 5.3	0.6 - 1.0	5.5 - 5.6	0.2 - 0.5
		Se	0.62 - 0.76	0.12 - 0.21	0.75 - 0.78	0.08 - 0.11	0.69 - 0.71	0.04 - 0.09
		Sp	0.80 - 0.89	0.09 - 0.17	0.80 - 0.83	0.08 - 0.11	0.84 - 0.85	0.02 - 0.06
Scenario 4 (mixed)	50	Cut-off	9.9 - 14.2	1.6 - 2.5	10.4 - 14.1	1.6 - 2.9	12.1 - 14.6	1.1 - 1.8
		Se	0.41 - 0.47	0.32 - 0.45	0.99 - 1.00	0.01 - 0.03	0.98 - 1.00	0.02 - 0.03
		Sp	0.65 - 0.82	0.22 - 0.24	0.97 - 0.99	0.03 - 0.04	0.97 - 0.98	0.03 - 0.06
	100	Cut-off	11.0 - 14.6	1.3 - 2.0	11.7 - 14.2	1.2 - 2.3	12.6 - 13.9	0.7 - 1.6
		Se	0.48 - 0.69	0.43 - 0.47	0.98 - 1.00	0.02 - 0.02	0.98 - 0.99	0.02 - 0.03
		Sp	0.79 - 0.94	0.13 - 0.23	0.97 - 0.98	0.02 - 0.04	0.97 - 0.97	0.02 - 0.05
	200	Cut-off	11.4 - 14.6	1.0 - 1.4	12.5 - 13.9	0.9 - 1.7	13.1 - 13.6	0.5 - 1.2
		Se	0.65 - 0.91	0.26 - 0.46	0.98 - 0.99	0.01 - 0.02	0.98 - 0.98	0.01 - 0.02
		Sp	0.90 - 0.97	0.04 - 0.16	0.96 - 0.98	0.02 - 0.03	0.96 - 0.97	0.01 - 0.03

For the 4 distributional scenarios and for total numbers of 50, 100 and 200 fictitious “individuals”, the ranges of mean values and SD values, found by varying P(D) from 0.1 to 0.9 in steps of 0.1, of optimal cut-off limits and sensitivities and specificities are reported. Mean values and SD values are based on 1000 repetitions each.

**Figure 2:**
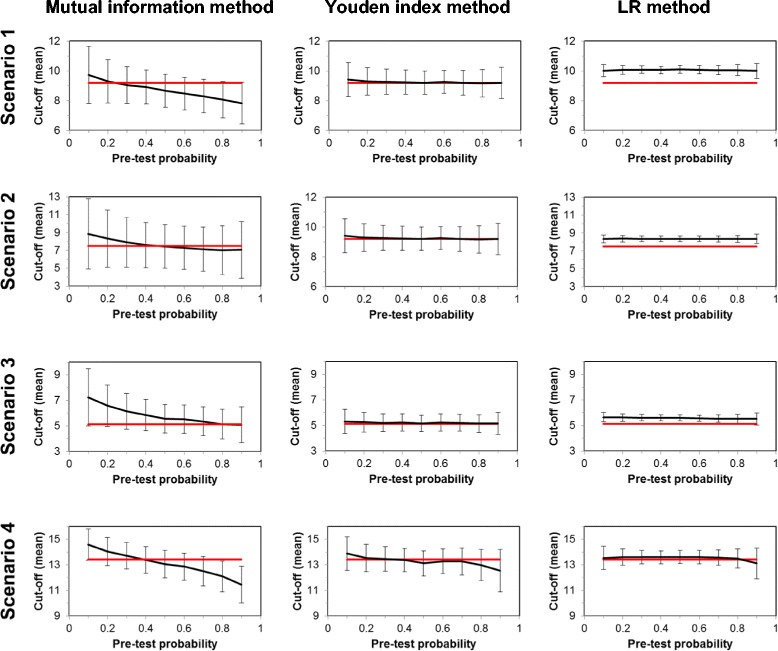
**Bias and precision of the cut-off levels by the three methods in dependence on pre-test probability P(D).** For the computations employing sample sizes of 200 fictitious “individuals”, the mean cut-off levels (based on 1000 repetitions each) detected by the three methods are shown together with their SD values in dependence on P(D) (black curves with error bars) and in comparison with the crossing points of the theoretical distribution functions of the two diagnostic categories (red horizontal lines).

For the mean cut-off values and their SD values obtained with each of the three methods, Figure 2 demonstrates for the four scenarios the dependence on P(D) as well as the deviations with respect to the theoretical crossing points of the distribution functions underlying the two diagnostic categories. (Notably, each result is based on 200 fictitious individuals and 1000 repetitions.)

Table 1 and Figure 2 reveal important and characteristic features of the results: first, while the Youden index method as well as the LR method produce cut-off levels which are remarkably stable with respect to the large variation of P(D), the Mutual information method yields, irrespective of the distributions used, monotonously decreasing cut-off levels with increasing P(D). So obviously this technique in the case of small P(D) optimizes specificity of the test, and with high P(D), sensitivity is optimized. Second, the Mutual information method is invariably associated with the largest SD values, followed by the Youden index method; the parametric LR method shows by far the smallest variations. On the other hand, the LR method tends to produce a constant positive bias; with the exception of Scenario 4 (strongly separated distribution functions underlying the two diagnostic categories) the cut-off levels found with this method lie consistently above the theoretical crossing points of the respective distribution functions. In fact, the smallest bias is found with the Youden index method; with the Mutual information method cut-off levels at small P(D) are generally too high, and too low with high P(D).

**Figure 3:**
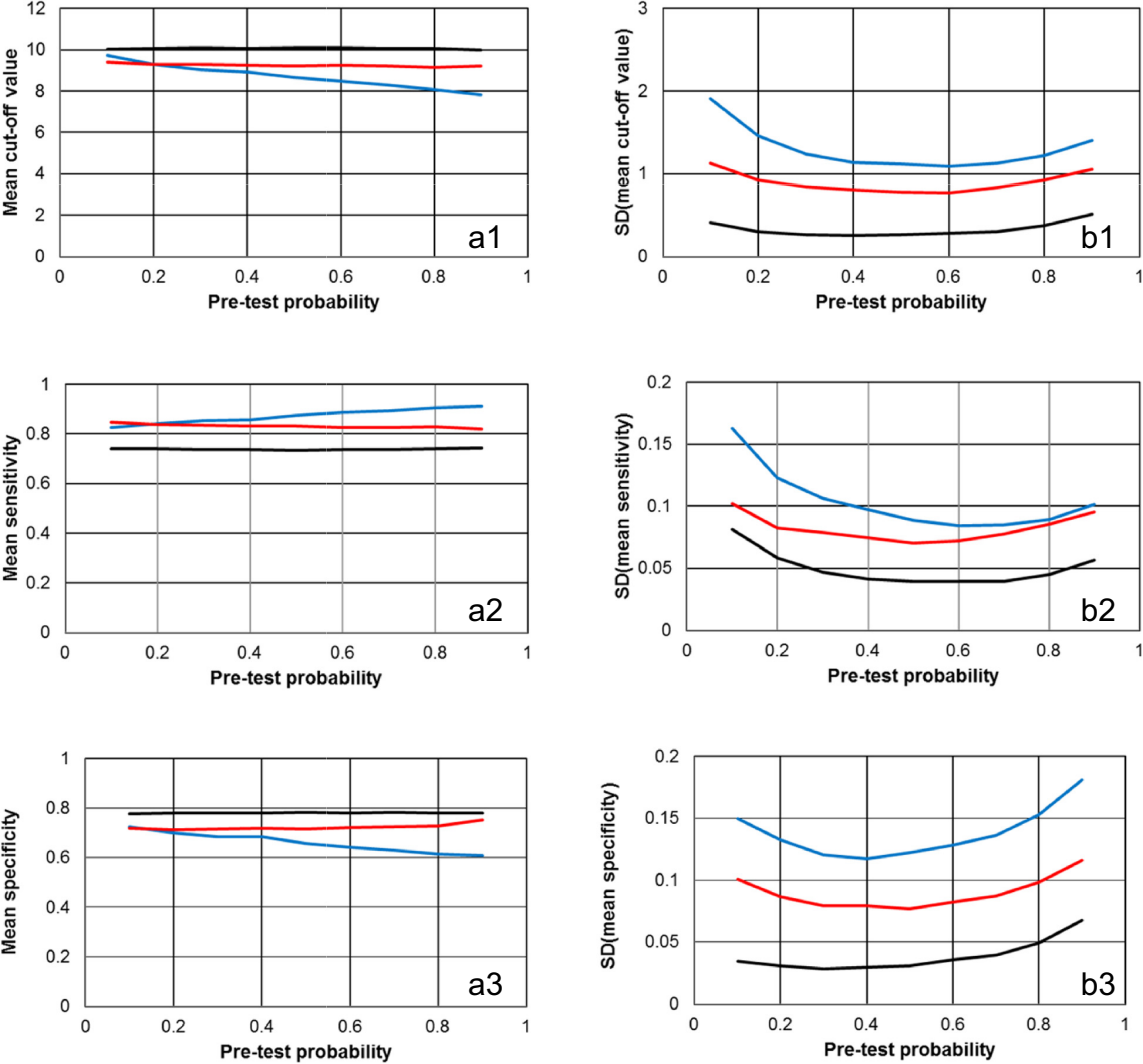
**The dependence of the cut-off levels and the associated sensitivities and specificities on P(D) for a representative example.** The results of the Monte-Carlo computations of scenario 1 with 200 fictitious “individuals” are shown in dependence on pre-test probability P(D). The left column **(panels a1, a2 and a3)** shows the mean values of cut-off levels and the associated sensitivities and specificities; the right column **(panels b1, b2 and b3)** visualizes the respective SD values. The colours of the lines symbolize the three methods for determining optimum cut-off levels: blue, Mutual information method; red, Youden index method; black, LR method.

Figure 3 visualizes in more detail the results obtained for Scenario 1 (lognormal distributions with mean value 2.0 and standard deviation 0.4 for the “healthy” category, and with mean value 2.5 and standard deviation 0.3 for the “diseased” category) and 200 fictitious “individuals” (N =200).

In accordance with Table 1 and Figure 2, the cut-off values and hence, the associated specificities, found by the LR method are usually slightly higher than those detected with the two non-parametric techniques. Consequently, the latter methods yield slightly better test sensitivities but slightly worse specificities than the LR method. As already shown in Figure 2 for the mean cut-off levels, also for sensitivities and specificities the stronger dependence of the Mutual information method on P(D) is clearly obvious from Figure 3 (panels a2 and a3). Analogously, also for the SD values of cut-off levels as well as of sensitivities and specificities the order LR method < Youden index method < Mutual information method is obtained.

Closer inspection of the SD results in Table 1 shows in addition that in accordance with expectation, the variances of the results decrease with increasing sample size N.

### Dependence on P(D) of the parametric estimates obtained by the LR-method

Despite the remarkable stability of the optimum cut-off thresholds as well as of sensitivities and specificities obtained by the parametric LR method over a broad range of P(D), the mean estimates of the logistic regression analyses (α0 and α1) nevertheless are somewhat dependent on P(D), and this dependence even remains after proper correction. For the example shown in Figure 3 [Scenario 1 (lognormal distributions with mean value 2.0 and standard deviation 0.4 for the “healthy” category, and with mean value 2.5 and standard deviation 0.3 for the “diseased” category) and 200 fictitious “individuals” (N =200)], at P(D) =0.10 the uncorrected mean intercept estimate (α0) is -5.246, and at P(D) =0.90 it increases to -3.075. Hence, the mean corrected intercept estimate decreases from -3.049 to -5.273 between these limits; and the mean slope estimates (α1) increases from 0.303 to 0.535. So in fact, also the corrected linear predictor functions (as well as the LR functions) change somewhat according to P(D). How strongly influence these dependencies the estimated post-test probabilities of disease? To answer this question, the mean values of the Monte-Carlo estimates of the logistic regression analyses at P(D) =0.10, 0.50 and 0.90 were used to calculate three respective corrected LR functions. With each of these three functions, then, according to the fact that the post-odds can be computed by multiplication of the pre-odds by the LR $\left[\frac{\mathrm{P}\left(\mathrm{D}\Big|\mathrm{x}\right)}{1\ \hbox{-}\ \mathrm{P}\left(\mathrm{D}\Big|\mathrm{x}\right)}=\frac{\mathrm{P}\left(\mathrm{D}\right)}{1\ \hbox{-}\ \mathrm{P}\left(\mathrm{D}\right)}\times \mathrm{L}\mathrm{R}\left(\mathrm{x}\right)\right]$, we compute the post-test probabilities P(D|x) (conditional probabilities for disease given test result x) as functions of test value x, again for three P(D) =0.10, 0.50 and 0.90.

**Figure 4:**
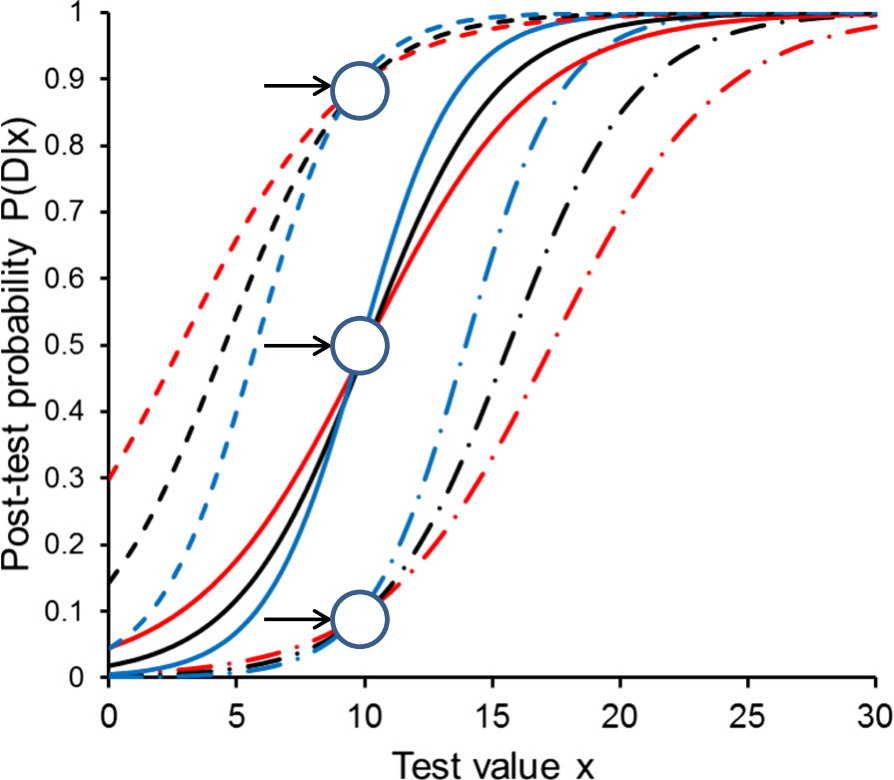
**The effect of P(D) on post-test probability functions obtained by the LR method.** Post-test probability functions P(D|x) of diagnostic value x, computed at different P(D) of 0.10 (dash-dotted lines), 0.50 (solid lines) and 0.90 (dashed lines). The three curves at each pre-test probability are obtained from the Monte Carlo experiment shown also in Figure 3 (scenario 1, 200 fictitious data sets) using the LR method, employing data sets with P(D) of 0.10 (red), 0.50 (black) and 0.90 (blue). Note that all three curves with the same colour run “parallel” to each other; i.e., they are obtained using the same slope parameter [mean value of the 1000 estimates for α1 at the respective P(D)]. The little arrows denote the crossing regions of the three curves obtained with the three different LR functions (see text for explanation).

Figure 4 shows the resulting curves of the post-test probabilities. Notably, if the corrected estimates of the logistic regression analyses were independent from P(D) of the data set employed, the three LR functions would coincide, and we would finally obtain only three different and parallel sigmoid curves (one for each P(D) used for the second step of this computation). However, as the estimated slope parameters (α1) increase with increasing P(D) in the data sets used for the logistic regression analyses, the sigmoidal post-test probability curves are steeper with respect to variation of test value x when we employ the estimates of logistic regression analyses obtained at higher P(D). Interestingly, each three curves obtained with the three different sets of logistic regression estimates for a specified P(D) in the second calculation step (in Figure 4 these are the curves with the same line style each) cross at a test value *x* ≈ 10 and at a post-test probability which approximates the actually specified P(D) (look at the little arrows in Figure 4).

Taking into account the results from Figure 2, we can easily understand this behaviour: Figure 3, panel a1, shows that with the LR method, a cut-off value of *x* ≈ 10 is obtained over the whole range of P(D). In other words, at *x* ≈ 10, all three different LR functions yield approximately unity, and hence, the post-test probabilities approximate P(D).

In addition we note, that for each of the three different LR functions the respective set of three curves at different P(D) in the second calculation step (in Figure 4 these are the curves with the same colour) shows “parallel” course, due to their common slope parameter α1 being representative for the respective LR function.

## Discussion

In this paper, we compare a parametric and two non-parametric methods of determining optimum binary cut-off levels of a ratio-scaled test using Monte Carlo technique. For scenarios with quite different distributional characteristics underlying the computer-generated data sets, and for different total numbers of fictitious “individuals” (i.e., data sets), we focus on the effects of varying P(D) on the optimum cut-off levels obtained, and on sensitivities and specificities associated with these threshold values.

Our study shows that the Youden index method and the LR method yield very stable mean cut-off levels over the whole range of P(D), while the results of the Mutual information method show a characteristic monotonous decrease of the mean cut-off values with increasing P(D). While the parametric LR method, based on logistic regression analysis followed by proper correction of the intercept parameter for P(D), produces by far the most precise estimates (smallest SD values), the method yields results which are positively biased for three of the four distributional scenarios studied. The best agreement between mean cut-off levels is obtained by the Youden index method, and the worst precision (largest SD values) is generally found by the technique of maximizing the mutual information statistic.

In perfect accordance with the behaviour of the mean optimum cut-off levels is the effect of the distributional scenarios as well as of P(D) on important test characteristics like sensitivities and specificities (and their SD values). Notably, as the LR technique generally produces the highest estimates of optimum cut-off levels (nearly irrespective of the distributional scenarios), it also yields the highest mean values for specificities, and in turn, the smallest mean values for sensitivities.

The estimates of the logistic regression analysis are somewhat dependent on the actual P(D) used, and post-test probability functions for the presence of disease, given a certain value of the diagnostic test variable, therefore show somewhat different slopes and positions; but as shown in Figure 4, the different curves obtained from logistic regression analyses with different P(D), when applied to compute post-test probabilities for a situation with an arbitrarily specified P(D) (in the second step), all cross approximately at a point in a P(D|x) vs. x diagram the abscissa of which is approximately equal to the optimum cut-off value and the ordinate of which approximates the specified P(D) (in the second step).

The results of the Monte Carlo simulations reported here appear to be representative for a broad variety of distributional characteristics underlying the test data in the “non-diseased” and the “diseased” category. Moreover, the results do not greatly vary when using 50, 100 or 200 fictitious “individuals”; clearly, the SD values obtained for increasing numbers of “individuals” are slightly decreasing. The study is restricted insofar as in any case, 1000 repetitions are employed for computing the respective mean values and SD values; however, this number is apparently high enough to guarantee quite stable estimates.

One might question the use of the crossing points of the involved distribution functions as the reference value for determining the bias of the methods: the crossing points of the distribution functions as used in our work imply a P(D) of 0.50 (both diagnostic categories would have the same weight) and, of course, varying the relative weights of the two distribution functions would lead to varying crossing points. For example, the theoretical crossing points for the distribution functions of scenario 4 vary between 15.748 and 11.358 when P(D) changes from 0.10 to 0.90; the reported value of 13.4333 is obtained for P(D) =0.50. However, we deliberately use the crossing points of the equally weighted distribution functions as the stable and correct reference value because we think that in diagnostic practice, the composition of a test sample with arbitrary P(D) should have as little effect as possible on critical results such as the optimum cut-off threshold. And in the light of these considerations, it is particularly surprising and satisfying that the Youden index method and the LR method indeed provide optimum cut-off value which are essentially independent from P(D).

In this work, we have concentrated on the specific influence of varying P(D) on few critical results of the diagnostic evaluation process; namely, the optimum cut-off levels and their associated sensitivities and specificities. We have not included many other important facets of modern test evaluation theory such as, e.g., utility aspects. It would certainly be promising to extend such simulation studies as our present one also on these and other advanced issues.

## Conclusions

Over a remarkably wide spectrum of distributional scenarios and over a wide range of different P(D) values, the Youden index method and the LR method give quite satisfactory results for optimum cut-off values in terms of stability and of test characteristics derived thereof. The results of the Mutual information method are stronger dependent on P(D) and, in addition, show the highest variation. Notably, the parametric LR technique yields particularly precise, however frequently positively biased results. A bonus of this method, on the other hand, is the straightforward transferability of the results to situations with other pre-test probabilities.

## Additional files

Additional file 1:
**The file “help.docx” explains how to use the MATHEMATICA notebooks supplied.**
Additional file 2:
**The MATHEMATICA notebook “monte_carlo_SDev.nb” performs the statistical calculations as well as the Monte-Carlo simulations and produces graphical output as well as results stored in EXCEL spreadsheets.**
Additional file 3:
**The MATHEMATICA notebook “distributions.nb” allows visualizing the distribution functions used for the two diagnostic categories and computes the respective crossing points.**

## References

[1] Linnet K, Bossuyt PMM, Moons KGM, Reitsma JB (2012). Quantifying the accuracy of a diagnostic test or marker. Clin Chem.

[2] Moons KGM, de Groot JAH, Linnet K, Reitsma JB, Bossuyt PMM (2012). Quantifying the added value of a diagnostic test. Clin Chem.

[3] Reitsma JB, Moons KGM, Bossuyt PMM, Linnet K (2012). Systematic reviews of studies quantifying the accuracy of diagnostic tests and markers. Clin Chem.

[4] Bossuyt PMM, Reitsma JB, Linnet K, Moons KGM (2012). Beyond diagnostic accuracy: the clinical utility of diagnostic tests. Clin Chem.

[5] Diamond GA, Hirsch M, Forrester JS, Staniloff HM, Vas R, Halpern SW, Swan HJC (1981). Application of information theory to clinical diagnostic testing. The electrocardiographic stress test. Circulation.

[6] Büttner J (1982). Grundlagen der Anwendung der Informationstheorie auf qualitative klinisch-chemische Untersuchungen. J Clin Chem Clin Biochem.

[7] Rudolph RA, Bernstein LH, Babb J (1988). Information induction for predicting acute myocardial infarction. Clin Chem.

[8] Kazmierczak SC, Catrou PG, Van Lente F (1995). Enzymatic markers of gallstone-induced pancreatitis identified by ROC curve analysis, discriminant analysis, logistic regression, likelihood ratios, and information theory. Clin Chem.

[9] Reibnegger G (2013). Beyond the 2×2-contingency table: a primer on entropies and mutual information in various scenarios involving **m** diagnostic categories and n categories of diagnostic tests. Clin Chim Acta.

[10] Albert A (1982). On the use and computation of likelihood ratios in clinical chemistry. Clin Chem.

[11] Birkett NJ (1988). Evaluation of diagnostic tests with multiple diagnostic categories. J Clin Epidemiol.

[12] Reibnegger G, Fuchs D, Hausen A, Werner ER, Werner-Felmayer G, Wachter H (1989). Generalization of the likelihood ratio concept for diagnostic tests with multiple diagnostic categories. J Clin Epidemiol.

[13] Reibnegger G, Fuchs D, Hausen A, Werner ER, Werner-Felmayer G, Wachter H (1989). Generalized likelihood ratio concept and logistic regression analysis for multiple diagnostic categories. Clin Chem.

