# Supplementary material for: Optimum binary cut-off threshold of a diagnostic test: comparison of different methods using Monte Carlo technique
Source: BMC Med Inform Decis Mak. 2014 Nov 25;14:99. doi: 10.1186/s12911-014-0099-1 (PMC4253606; doi:10.1186/s12911-014-0099-1)
Supplement: Additional file 1: — The file “help.docx” explains how to use the MATHEMATICA notebooks supplied. [file 12911_2014_99_MOESM1_ESM.docx]

Use of the MATHEMATICA files:

1. **distributions.nb**

This notebook produces the graphical representation of the distribution functions used. Moreover, for each scenario, it computes the crossing points of the distribution functions used for the non-diseased and the diseased individuals.

In the file, you find the lines

**a=PDF[LogNormalDistribution[2.0,.40],x];**

**b=PDF[LogNormalDistribution[2.5,.30],x];**

with the crossing point x = 9.20041 and the picture

By changing the definitions for a and b you can compute the crossing points for varying P(D): For example, a P(D) of 0.20 would lead to (scenario 1)

**a=0.80 * PDF[LogNormalDistribution[2.0,.40],x];**

**b=0.20 * PDF[LogNormalDistribution[2.5,.30],x];**

with the crossing point x = 13.6149. The corresponding picture would look as follows:


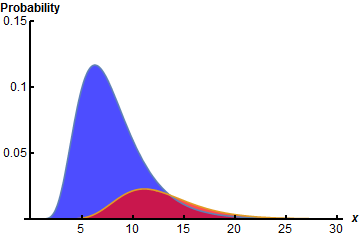


can be obtained by changing the first plot-paragraph into

$$\boldsymbol{Plot[\{}\text{.8}\mathbf{*}\mathbf{PDF[LogNormalDistribution[2.,}\text{.40}\mathbf{],}\mathbf{x}\mathbf{],}\text{.2}\mathbf{*}\mathbf{PDF[LogNormalDistribution[2.5,}\text{.30}\mathbf{],}\mathbf{x}\boldsymbol{]\},\{}\mathbf{x}\boldsymbol{,0,30\},Filling\to\{1\to\{Axis,Directive[Opacity[0.7],Blue]\},2\to\{Axis,Directive[Opacity[0.7],Red]\}\},PlotRange\to\{0,}\text{.15}\boldsymbol{\},Ticks\to\{\{5,10,15,20,25,30\},\{0,0.05,0.1,0.15\}\},TicksStyle\to Directive[Black,12],AxesLabel\to\{Style[}\mathbf{x}\boldsymbol{,Medium,Bold,Black],Style[Probability,Medium,Bold,Black]\},AxesStyle\to\{Directive[Thick,Black],Directive[Thick,Black]\}]}$$

Notably, however, as explained in the Discussion section, in our manuscript we have deliberately used only the crossing points of the “undisturbed” distributions (according to P(D) = 0.50) as reference points for defining the “bias” of the resulting cut-off values.

1. **monte_carlo_SDev.nb**

This is the main notebook doing the calculations as well as the Monte Carlo simulation. For the user, the part

**Parameter input for the analysis**

**ntotal=50; ngesundvec={45, 40, 35, 30,25, 20, 15, 10, 5}; maxsamples=1000;**

**(* ntotal=20; ngesundvec={2,10}; maxsamples=2; *)**

**myAnalyseModule@@@{**

**{LogNormalDistribution[2.0,.40],LogNormalDistribution[2.5,.30]}**

**};**

is the relevant one. **ntotal** is the number of studied fictitious individuals, and the vector **ngesundvec** defines the computational steps for the various P(D) values. In the example we have **ntotal = 50** and the numbers **45, 40, …** and so on in **ngesundvec** mean P(D) values of 0.10 (45 non-diseased and 5 diseased subjects), 0.20 (40 non-diseased and 10 diseased subjects) and so on. **maxsamples** is the number of Monte Carlo-repetitions of the experiments.

The second part defines the distributions for the non-diseased and the diseased subjects.

**Scenario 1:**

**LogNormalDistribution[2.0,.40],LogNormalDistribution[2.5,.30]**

**Scenario 2:**

**ChiSquareDistribution[7.],ChiSquareDistribution[10.]**

**Scenario 3:**

**InverseGammaDistribution[6.,20],InverserGammaDistribution[3.,20]**

**Scenario 4:**

**ChiSquareDistribution[6.],WeibullDistribution[10.,20.]**

The notebook produces 7 result files:MyResultPLot_1.PDF, MyResultPLot_1.TIF, MyResultPLotsSDev_1.PDF and MyResultPLotsSDev_1.TIF are figures showing the P(D)-dependence of the mean values of the cut-off thresholds, sensitivities and specificities as well as the associated standard deviations of the Monte-Carlo-experiments in dependence on P(D) and the method used. Note that in these result files there are also the results obtained when proper correction of the LR technique according to the pre-test odds is left out; these results (method “P(D+|T+)=0.5”) are not reported in the paper. Also important: these figures are not exactly the same as shown in the manuscript.

The remaining three files give the results in terms of numerical results stored in EXCEL documents which are self-explaining. Again, the results listed under “Ppost = 0.5” are not reported in the manuscript. The Figures in the manuscript were constructed using basically these results; however, there was some additional work necessary to produce the final figures for the manuscript.

All relevant computations are done in the paragraphs Computations 1 and Computations 2. These may be viewed by clicking on the respective brackets on the right side of the notebook.
